# Exploring Prenatal Signs of Congenital Lipomatous Overgrowth, Vascular Malformations, Epidermal Nevi, and Skeletal Anomalies (CLOVES) Syndrome: A Case Report and Literature Review

**DOI:** 10.7759/cureus.88455

**Published:** 2025-07-21

**Authors:** Emily Henuzet, Laurence Boon, Dana Dumitriu, Leentje Peetermans, Patricia Steenhaut

**Affiliations:** 1 Department of Obstetrics, Université Catholique de Louvain, Brussels, BEL; 2 Department of Plastic Surgery, Université Catholique de Louvain, Brussels, BEL; 3 Department of Pediatric Radiology, Université Catholique de Louvain, Brussels, BEL; 4 Department of Neonatalogy, Université Catholique de Louvain, Brussels, BEL

**Keywords:** antenatal diagnosis, cloves syndrome, pik3ca mutation, pros, segmental overgrowth, vascular malformations

## Abstract

We report a rare case of partial prenatal diagnosis of congenital lipomatous overgrowth, vascular malformations, epidermal nevi, and skeletal anomalies (CLOVES) syndrome in a fetus presenting extensive dorsal lymphatic malformation, bilateral polydactyly and syndactyly, hypertrophy on the left foot, and suspected cryptorchidism. Amniocentesis with comparative genomic hybridization (CGH) and trio-exome sequencing did not reveal any pathogenic variant. Postnatal clinical examination and imaging confirmed the malformations, including a multilocular macrocystic lymphatic malformation with retroperitoneal extension. Sirolimus therapy was initiated, resulting in a modest reduction in the volume of the dorsal mass within the first two months of treatment. This report underscores key prenatal features that may raise suspicion for CLOVES syndrome, helping clinicians to differentiate it from other overgrowth disorders such as Proteus syndrome and conditions within phosphatidylinositol-4,5-bisphosphate 3-kinase catalytic subunit alpha (PIK3CA)-related overgrowth spectrum (PROS), supporting improved diagnosis and counseling during pregnancy.

## Introduction

Congenital lipomatous overgrowth, vascular malformations, epidermal nevi, and skeletal anomalies (CLOVES) syndrome is a rare, sporadic overgrowth disorder caused by a post-zygotic mutation in the phosphatidylinositol-4,5-bisphosphate 3-kinase catalytic subunit alpha (PIK3CA) gene [1]. This somatic mutation results in segmental overgrowth, complex vascular malformations, and musculoskeletal anomalies.

As part of the PIK3CA-related overgrowth spectrum (PROS), CLOVES syndrome presents with highly heterogeneous clinical features, often overlapping with other overgrowth disorders, which complicate prenatal diagnosis. Although molecular testing can confirm the diagnosis postnatally, antenatal genetic confirmation is often inconclusive due to the mosaic distribution of the mutation.

While approximately 200 cases of CLOVES syndrome have been reported in the literature, this is only the fourth case described with prenatal findings. Given the potential for severe congenital anomalies and long-term morbidity, earlier recognition of suggestive prenatal features is essential. This article presents a case of partial antenatal diagnosis and reviews key imaging findings that may support early identification and targeted counseling during pregnancy.

## Case presentation

We report the case of a 31-year-old woman, gravida 5 para 4, with a history of two vaginal deliveries and two cesarean sections. She had no relevant medical or surgical history. The current pregnancy, conceived with a new partner, was initially followed at a peripheral healthcare facility.

A non-invasive prenatal test (NIPT), routinely performed in Belgium, indicated a male fetus with no chromosomal abnormalities. However, a second-trimester ultrasound revealed subcutaneous edema and a structural anomaly affecting one foot, prompting referral to our tertiary-level fetal medicine center for further evaluation.

Antenatal Investigations

**Figure 1:**
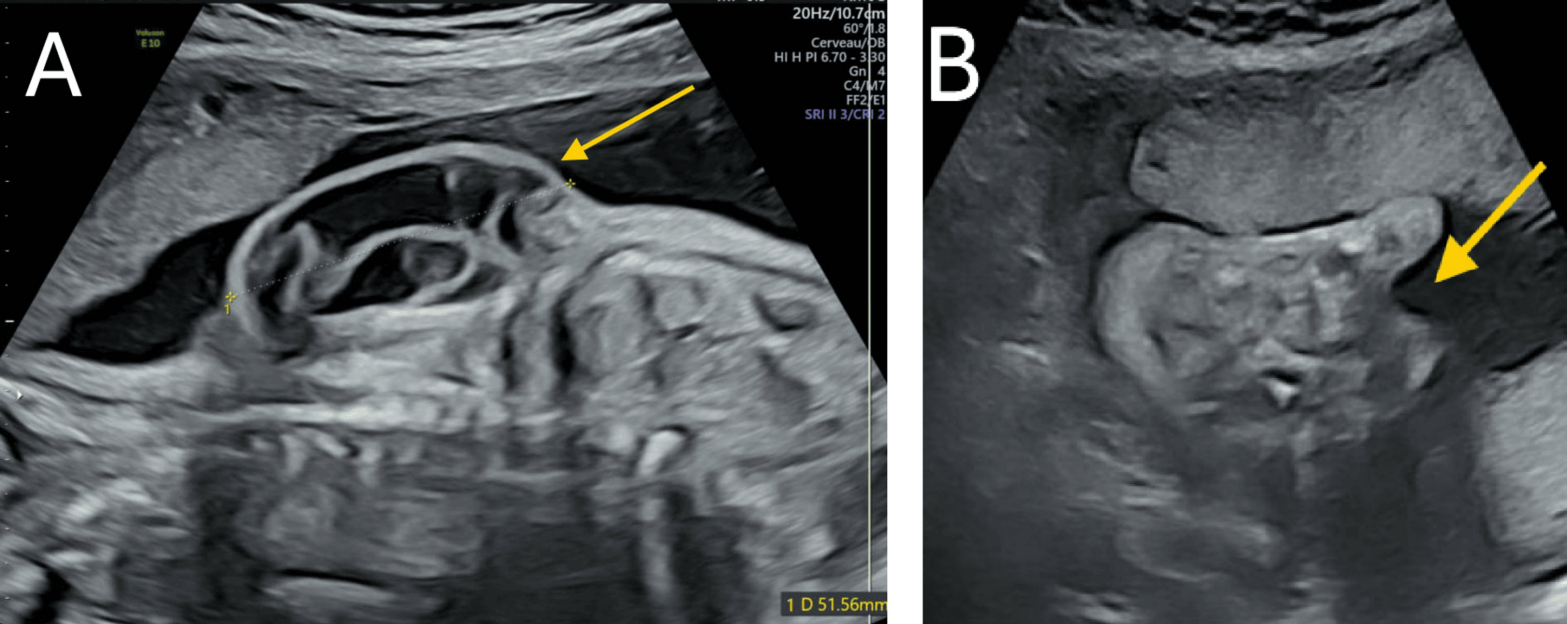
Fetal ultrasound images at 22 weeks and six days of gestation A: posterior subcutaneous fluid-filled mass (arrow) along the thoracic spine; B: sandal gap (arrow)

A fetal ultrasound performed at 22 weeks and six days of gestation revealed a eutrophic male fetus with an estimated weight of approximately 600 g (73rd percentile) and normal vitality. However, several anomalies were identified, such as a prominent, septated posterior subcutaneous fluid-filled mass along the thoracic spine (Figure 1A). No vertebral defects were detected, although an underlying medullary abnormality could not be excluded. Additionally, the right foot exhibited six metatarsals, plantar edema, and a sandal gap (Figure 1B).

Amniocentesis was performed the same day. Comparative genomic hybridization (CGH) array and trio-exome sequencing showed no pathogenic variants. However, large regions of homozygosity were observed, suggesting potential consanguinity, although unrelated to the identified anomalies.

**Figure 2:**
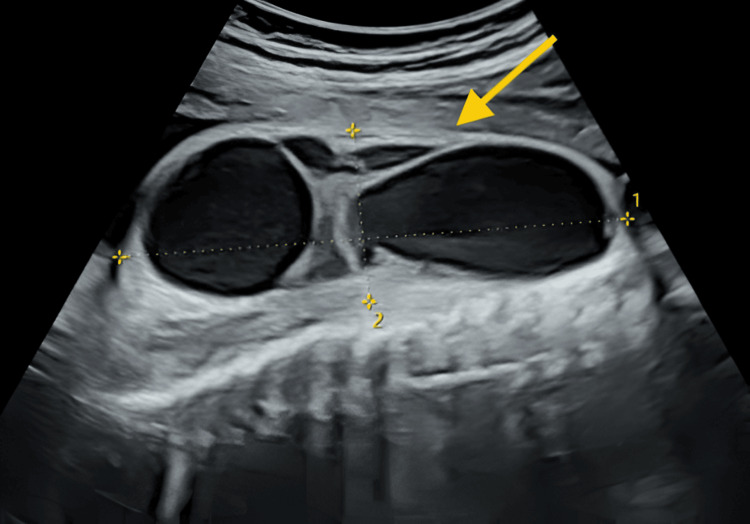
Fetal ultrasound image of the posterior subcutaneous cystic mass measuring 98 × 76 × 33 mm at 33 weeks of gestation

Monthly follow-up ultrasounds confirmed persistence of the initial findings and revealed additional malformations. A progressive enlargement of the posterior cystic mass, which measured 98 × 76 × 33 mm at 33 weeks of gestation (Figure 2). The lesion was multilocular and septated, with no detectable vascular flow on Doppler imaging. The vertebrae appeared normal, though an associated spinal cord anomaly could not be definitively ruled out. Subsequently, a broad right foot consisted of postaxial polydactyly and a sandal gap or ectrodactyly, though limb mobility was preserved. On the other hand, the left foot showed suspected postaxial polydactyly and possible syndactyly between the second and third toes, with persistent hyperextension of the digits. Finally, there was also a suspected left-sided cryptorchidism.

Despite these abnormalities, fetal Doppler assessments and vitality remained within normal limits throughout gestation.

At 32 weeks of gestation, the case was reviewed by a multidisciplinary vascular anomalies team for diagnostic clarification and management planning. The constellation of findings - including the extensive dorsal macrocystic lymphatic malformation and foot anomalies - suggested a slow-flow vascular malformation syndrome such as CLOVES or another condition within the PROS. Given our institutional experience with in utero administration of sirolimus in cases of life-threatening lymphatic malformation [2], maternal therapy was considered. However, as the lesion did not present an immediate risk to fetal well-being, antenatal treatment was not pursued. Fetal magnetic resonance imaging (MRI) was likewise deferred for the same reason.

A targeted gene panel for lymphovascular malformations was also performed and yielded normal results. The parents received multidisciplinary counseling, including input from the fetal medicine and genetics teams, and opted to continue the pregnancy.

Obstetric and Neonatal Management

An elective cesarean section was performed at 37 weeks and six days of gestation. The male infant weighed 2,945 g (34th percentile), measured 47 cm in length (18th percentile), and had a head circumference of 32.5 cm (18th percentile). Apgar scores were 9, 9, and 10 at 1, 5, and 10 minutes, respectively.

**Figure 3:**
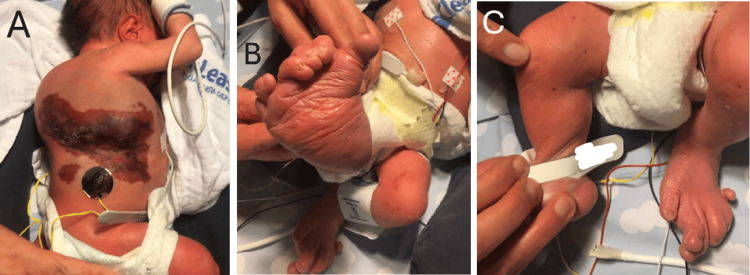
Images of the malformations at birth A: Thoracodorsal capillary-lymphatic malformation involving the skin and the subcutaneous tissue. B: Hypertrophy, postaxial polydactyly, and syndactyly of the right foot. C: Polydactyly and syndactyly of the left foot.

Postnatal examination confirmed the presence of an extensive thoracodorsal capillary-lymphatic malformation involving the skin and subcutaneous tissues (Figure 3A). Malformations of both feet were also observed, including hypertrophy, postaxial polydactyly, and syndactyly (Figures 3B-3C). Bilateral cryptorchidism was noted on clinical examination.

Postnatal Assessment and Evolution

**Figure 4:**
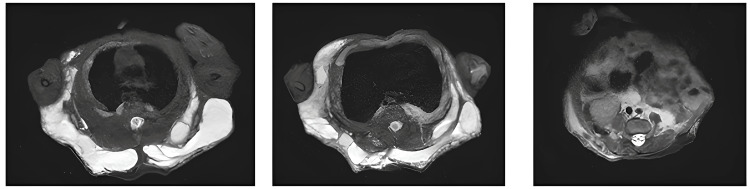
Day 10 post-natal MRI centered on the lymphatic malformation Axial T2 fat sat views of the macrocystic lymphatic malformation of the posterior wall with subcutaneous, muscular, and profound involvement (left pleural, retroperitoneal).

**Figure 5:**
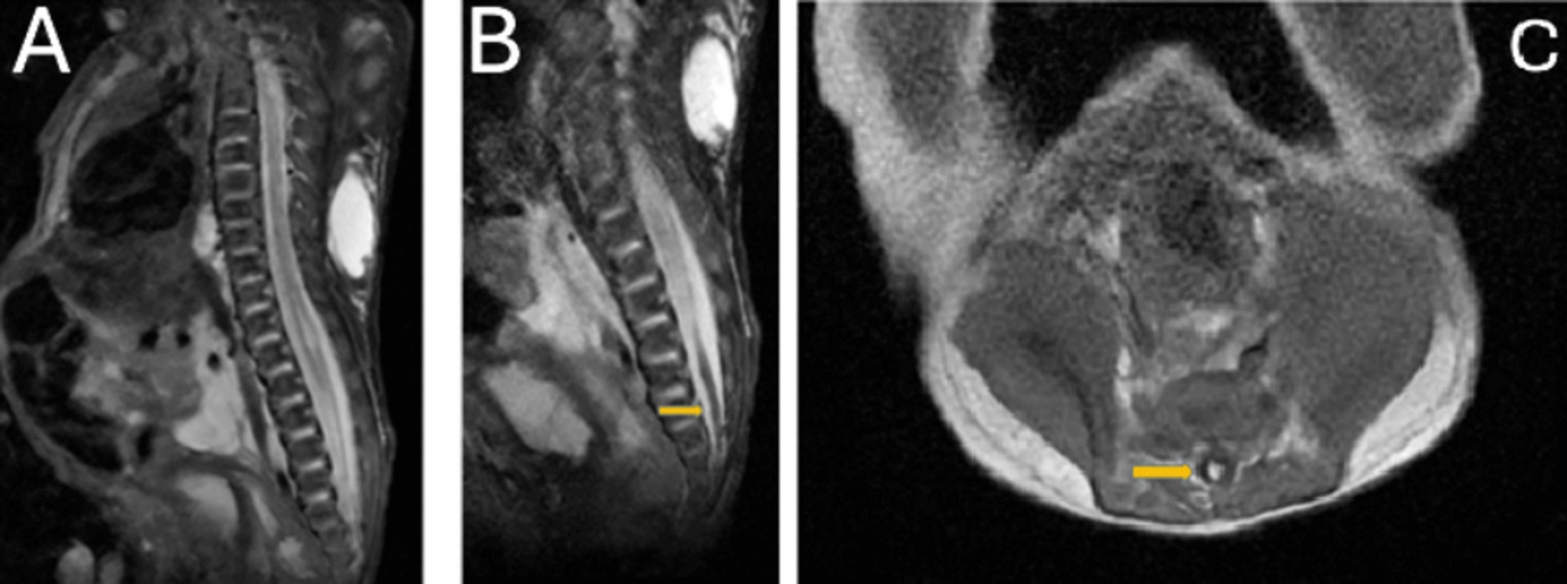
Day 10 post natal MRI centered on the anomalies of the spinal cord A and B: Sagittal T2 fat-saturated views of the syringomyelic cavity of the spinal cord in the lumbar region and an attached cord with lipoma of the filum terminale (arrow). C: Axial T1 view of the filum terminale lipoma (arrow).

Postnatal imaging, including Doppler ultrasound and thoracoabdominopelvic MRI, confirmed the presence of a large subcutaneous thoracodorsal macrocystic lymphatic malformation. The lesion extended into the muscular planes, the left pleural cavity, and the retroperitoneal space (Figure 4). Spinal MRI revealed a syringomyelic cavity in the lumbar spinal cord, along with a tethered cord and a lipoma of the filum terminale (Figure 5). Transfontanellar ultrasound findings were normal. Doppler imaging of the lower extremities excluded deep venous anomalies, including persistence of the embryonic vein of Servelle - frequently associated with Klippel-Trenaunay syndrome.

**Figure 6:**
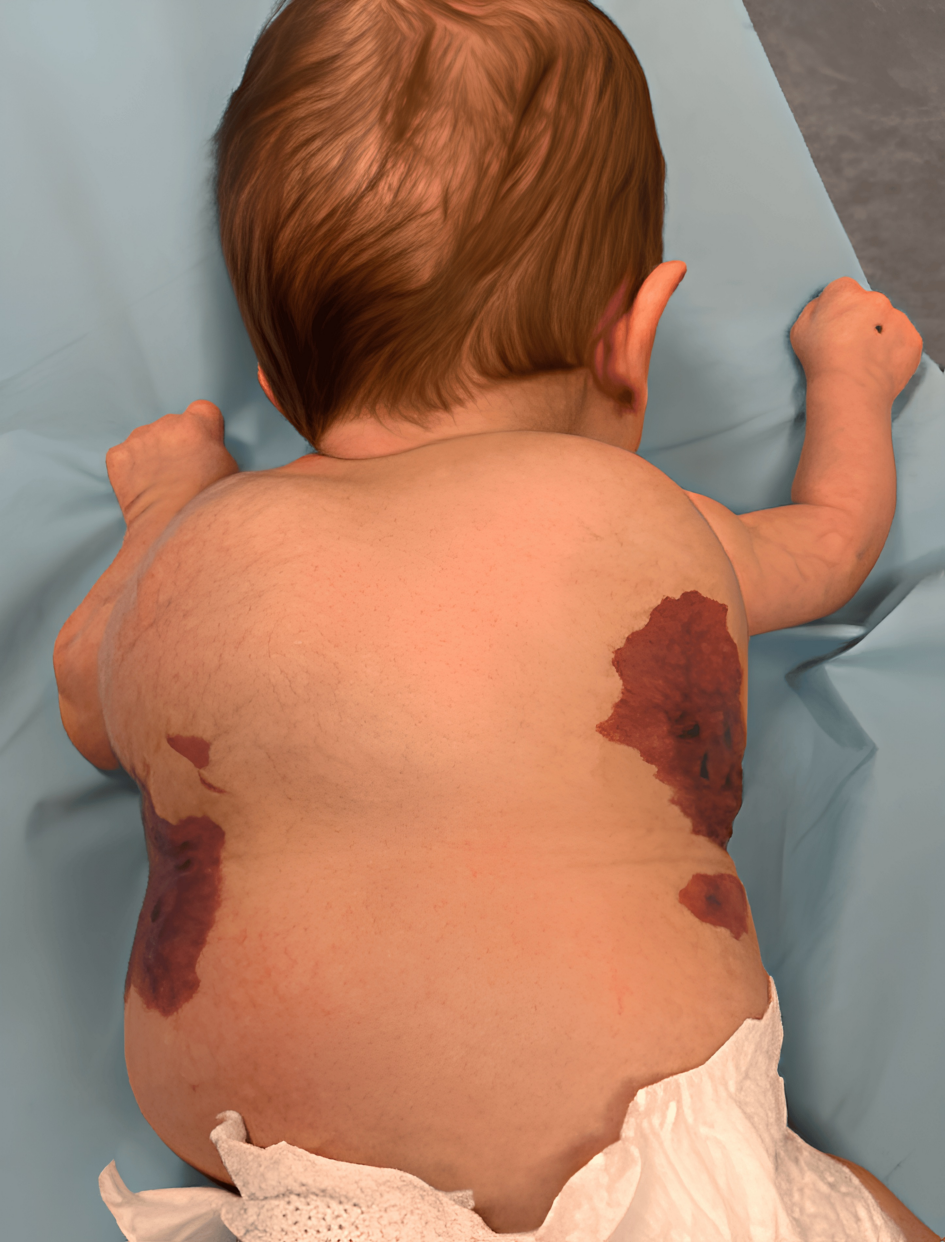
Improvement in cutaneous appearance and reduced erythema at two months of follow-up

Given the extent of the lymphatic malformation, oral sirolimus therapy was initiated on day three of life. At the two-month follow-up, a slight reduction in lesion volume was noted, along with improvement in cutaneous appearance and reduced erythema (Figure 6). Growth parameters remained within normal limits, and sirolimus was well tolerated without any adverse events. Persistent hypertrophy of the right foot warranted orthopedic follow-up at six months of age. Bilateral intra-abdominal testes were confirmed via urological ultrasound, requiring continued monitoring by pediatric urology.

Due to the lack of improvement in hypertrophy, treatment with alpelisib, a PI3Kalpha-selective inhibitor, was initiated at six months of age with the goal of modulating PIK3CA-driven tissue overgrowth.

## Discussion

Definition

CLOVES syndrome is a rare, complex, non-hereditary overgrowth disorder first described by Sapp et al. [3] in 2007 as a novel hypertrophic condition initially referred to as "CLOVE syndrome." Following the identification of skeletal anomalies, particularly spinal deformities such as scoliosis, the acronym was revised by Alomari [4] in 2009 to "CLOVES" - representing congenital lipomatous overgrowth, vascular malformations, epidermal nevi, and scoliosis/skeletal/spinal anomalies.

Genetics

CLOVES syndrome is part of the PIK3CA-related overgrowth spectrum, caused by somatic, post-zygotic activating mutations in the PIK3CA gene [5]. This gene encodes the catalytic subunit of phosphatidylinositol-3-kinase (PI3K), a key enzyme in the PI3K/AKT/mTOR signaling pathway, which regulates cellular growth, proliferation, and survival. Constitutive activation of this pathway leads to segmental tissue overgrowth, primarily affecting mesodermal derivatives (adipose tissue, vascular and lymphatic systems, skeletal muscle and bone), as well as ectodermal components such as skin and neuroconnective tissue.

The mosaic distribution of the mutation explains the segmental, asymmetric, and localized nature of the overgrowth and associated malformations.

Epidemiology

With approximately 200 cases of CLOVES syndrome reported in the literature to date, the estimated incidence is below one in 1,000,000 live births. The condition affects both sexes and has been described in all racial populations. Clinical manifestations are typically congenital and appear either in utero or within the first year of life [5], although diagnostic recognition may be delayed in milder forms.

Overgrowth syndrome entity

CLOVES syndrome belongs to a broader group of mosaic overgrowth syndromes caused by postzygotic mutations, often in genes involved in growth-regulatory pathways. The 2014 National Institutes of Health (NIH) consensus defined PROS as a distinct clinical-genetic entity encompassing a spectrum of phenotypes caused by PIK3CA mutations [6]. This group includes fibroadipose overgrowth (FAO), hemihyperplasia-multiple lipomatosis (HHML), dysplastic megalencephaly (DMEG), megalencephaly-capillary malformation (MCAP), CLOVES, and Klippel-Trenaunay syndrome (KTS), added in 2016 due to overlapping vascular and overgrowth features.

Treatments

Currently, there is no curative treatment for CLOVES syndrome. Given the multisystem involvement and the complexity of the associated malformations, optimal management requires a multidisciplinary approach, involving specialists in vascular anomalies, dermatology, orthopedics, plastic surgery, and pediatric urology.

The discovery that PIK3CA mutations activate the PI3K/AKT/mTOR pathway, which also plays a pivotal role in oncogenesis, has opened the door to repurposing targeted therapies initially developed for cancer. Inhibitors of this pathway - including sirolimus (rapamycin), everolimus, and other allosteric mTOR inhibitors - have been used with some efficacy in managing vascular malformations and overgrowth syndromes [7,8].

More recently, PIK3CA-selective inhibitors such as alpelisib have shown promise in clinical trials for PROS-related disorders. Several ongoing studies are evaluating the safety and efficacy of these agents in both pediatric and adult populations [6,9]. However, long-term data on functional outcomes, growth, and tolerability remain limited.

Prenatal diagnosis

Despite growing recognition of CLOVES syndrome, prenatal diagnosis remains extremely rare. The mosaic nature of PIK3CA mutations complicates genetic confirmation in utero, and many cases go undetected until postnatal presentation.

In 2007, Sapp et al. [3] described seven patients with CLOVES, of whom five had retrospective documentation of prenatal anomalies. These included subcutaneous fluid collections, septated cystic masses, ventriculomegaly, clubfoot, and sonographic features, suggestive of abdominal wall defects or placental chorioangiomas - though none led to a formal prenatal diagnosis at the time.

Fernandez-Pineda et al. [10] reported the first prenatal suspicion of CLOVES in a dizygotic twin, based on a multicystic abdominal mass and facial asymmetry. Subsequent reports by Puvabanditsin et al. [11] and Emrick et al. [12] described fetuses with truncal lymphatic malformations, asymmetric limb hypertrophy, and subcutaneous septated cystic lesions. Only the latter case had prenatal molecular confirmation of CLOVES via detection of a 38% PIK3CA mosaicism in amniocytes.

In our case, prenatal sonographic findings were suggestive, including a large dorsal macrocystic lymphatic malformation, limb malformations, and cryptorchidism. However, genetic investigations - including CGH array, trio-exome sequencing, and a targeted vascular malformation panel - were inconclusive, underscoring the diagnostic limitations in mosaic disorders.

Table 1 provides a comparative overview of these rare prenatally suspected cases, highlighting recurrent imaging features such as abdominal or dorsal cystic masses, limb asymmetry, and polydactyly.

**Table 1:** Comparative summary of published cases of CLOVES syndrome with prenatal findings CLOVES: Congenital Lipomatous Overgrowth, Vascular Malformations, Epidermal Nevi, and Skeletal Anomalies; MRI: Magnetic Resonance Imaging, CGH: Comparative Genomic Hybridization

Case	Fernandez et al. [10] (2010)	Puvabanditsin et al. [11] (2014)	Emrick et al. [12] (2014)	Present case
Maternal age	Not specified	39 years	Not specified	31 years
Parity	Not specified	G2P1	G2P0	G5P4
Antenatal ultrasound findings	Septated subcutaneous fluid collections in the abdominal wall, cerebral and facial asymmetry	Cystic hygroma, abdominal subcutaneous fluid collections with septations	Multifocal cystic malformations of the trunk (vascular/lymphatic), left leg and foot, asymmetric hypertrophy of the left leg and foot, polyhydramnios	Thoracodorsal macrocystic lymphatic malformation, bilateral foot anomalies (polydactyly, sandal gap, hypertrophy), suspected cryptorchidism
Gestational age at first abnormal ultrasound	26 weeks	18 weeks	26 weeks	22 weeks
Fetal MRI	Abdominal macrocystic lymphatic malformation	Not performed	Lymphatic malformation and asymmetric foot hypertrophy	Not performed
Obstetric complications	Dizygotic twin pregnancy	Gestational diabetes	Polyhydramnios with amnioreduction	None
Genetic studies	Normal karyotype (46, XX)	Not reported	Normal karyotype and microarray, 38% PIK3CA mosaicism in amniocytes	Normal CGH array, trio-exome sequencing, and vascular malformation panel

The clinical features of overgrowth syndromes frequently overlap, contributing to significant diagnostic uncertainty. Retrospective analyses suggest that findings described in earlier case reports of Klippel-Trenaunay or Proteus syndrome may, in fact, be more consistent with what is now recognized as CLOVES syndrome.

Table 2 outlines key antenatal features that help distinguish CLOVES syndrome from other disorders within the PROS. CLOVES is typically characterized by truncal vascular malformations, skeletal anomalies, limb asymmetry, polydactyly, and lipomatous overgrowth - features that may facilitate prenatal recognition when present in combination.

**Table 2:** Prenatal features of CLOVES syndrome compared with other overgrowth syndromes CLOVES: Congenital Lipomatous Overgrowth, Vascular Malformations, Epidermal Nevi, and Skeletal Anomalies

Syndrome	Genetic cause	Main prenatal features	Vascular malformations	Limb overgrowth	Neurological anomalies	Polydactyly/ Syndactyly	Diagnostic challenges
								CLOVES	PIK3CA (somatic mutation)	Truncal macrocystic lymphatic malformation, limb asymmetry, foot anomalies (polydactyly, hypertrophy), possible cryptorchidism	Frequent (capillary-lymphatic, slow-flow)	Segmental, asymmetric	Rare (e.g., tethered cord)	Frequent (especially postaxial)	Variable expression; mosaicism limits molecular detection
Fibroadipose Overgrowth/ Hemihyperplasia-Multiple Lipomatosis [13]	PIK3CA (somatic)	Subtle limb or body asymmetry; soft tissue overgrowth	Absent or minimal	Yes, usually soft tissue only	Absent	Rare	Difficult to detect antenatally; often mild features
Megalencephaly-Capillary Malformation/ Dysplastic Megalencephaly [13]	PIK3CA (somatic)	Megalencephaly, ventriculomegaly, abnormal cortical development	Possible (mainly capillary)	Possible	Frequent (polymicrogyria, ventriculomegaly)	Occasional	More readily diagnosed on fetal neuroimaging
Klippel-Trenaunay [14]	Unknown; possibly PIK3CA (in mosaic form)	Limb asymmetry, subcutaneous edema, enlarged extremities	Present (capillary-lymphatic-venous), risk of Servelle vein persistence	Yes	Rare	Rare	May mimic CLOVES; prenatal Doppler may reveal the Servelle vein
Proteus [15]	AKT1 (somatic)	Usually undetectable prenatally; postnatal progressive overgrowth	Infrequent	Yes, progressive and disorganized	Occasional (e.g., hemimegalencephaly)	Rare	Typically, not diagnosed prenatally; variable expressivity

In contrast, other PROS conditions, such as FAO and HHML, primarily manifest as asymmetric soft tissue overgrowth or hemihypertrophy, without associated vascular malformations [13]. These subtler features are more difficult to detect in utero.

DMEG and MCAP are characterized by megalencephaly and cerebral anomalies, sometimes accompanied by vascular malformations or polydactyly. While overlapping features with CLOVES syndrome may exist, DMEG and MCAP typically present with a more pronounced neurological phenotype.

KTS shares certain features with CLOVES, including limb hypertrophy and vascular malformations [14]. However, it is often associated with systemic complications related to the persistence of the embryonic vein of Servelle, which can result in pulmonary embolism if not identified and treated early.

Proteus syndrome, associated with AKT1 mutations, is less commonly detected prenatally, as its hallmark progressive asymmetric overgrowth usually becomes apparent during childhood [15]. Nonetheless, isolated cases of hemimegalencephaly have been reported in utero.

Given the phenotypic overlap among these syndromes, accurate prenatal diagnosis requires detailed ultrasonographic assessment in specialized centers, the identification of distinguishing features, and, when possible, molecular confirmation to guide perinatal management and postnatal surveillance.

## Conclusions

CLOVES syndrome is a rare and complex overgrowth syndrome disorder that presents a significant diagnostic challenge during the prenatal period due to its phenotypic overlap with other syndromes in the PROS. Although antenatal detection remains exceptional, certain sonographic findings - such as large septated cystic masses, limb asymmetry, and polydactyly - should raise clinical suspicion and prompt referral to a specialized fetal medicine unit.

This case adds to the limited number of reports describing CLOVES syndrome with prenatal features and highlights the value of detailed fetal imaging and multidisciplinary evaluation. While standard genetic testing methods fail to detect low-level mosaicism prenatally, emerging targeted therapies, including PI3K inhibitors such as sirolimus or alpelisib, are offering new therapeutic avenues in the postnatal setting. Improved awareness of characteristic prenatal signs, combined with collaborative diagnostic strategies and advances in molecular techniques, may enhance early recognition of PROS disorders and inform perinatal management and long-term follow-up.
